# Oxidative Stress and Respiratory Diseases in Preterm Newborns

**DOI:** 10.3390/ijms222212504

**Published:** 2021-11-19

**Authors:** Laura Cannavò, Serafina Perrone, Valeria Viola, Lucia Marseglia, Gabriella Di Rosa, Eloisa Gitto

**Affiliations:** 1 Neonatal and Pediatric Intensive Care Unit, Department of Human Pathology of the Adult and Developmental Age “Gaetano Barresi”, University of Messina, 98125 Messina, Italy; laura_cannavo@hotmail.it (L.C.); valeriaviola89@gmail.com (V.V.); lmarseglia@unime.it (L.M.); egitto@unime.it (E.G.); 2 Neonatology Unity, Department of Medicine and Surgery, University of Parma, 43126 Parma, Italy; 3 Unit of Child Neurology and Psychiatry, Department of Human Pathology of the Adult and Developmental Age “Gaetano Barresi”, University of Messina, 98125 Messina, Italy; gdirosa@unime.it

**Keywords:** oxidative stress, preterm, bronchopulmonary dysplasia, respiratory distress syndrome, pulmonary hypertension of the newborn

## Abstract

Premature infants are exposed to increased generation of reactive oxygen species, and on the other hand, they have a deficient antioxidant defense system. Oxidative insult is a salient part of lung injury that begins as acute inflammatory injury in respiratory distress disease and then evolves into chronic and structural scarring leading to bronchopulmonary dysplasia. Oxidative stress is also involved in the pathogenesis of pulmonary hypertension in newborns through the modulation of the vascular tone and the response to pulmonary vasodilators, with consequent decrease in the density of the pulmonary vessels and thickening of the pulmonary arteriolar walls. Oxidative stress has been recognized as both a trigger and an endpoint for several events, including inflammation, hypoxia, hyperoxia, drugs, transfusions, and mechanical ventilation, with impairment of pulmonary function and prolonged lung damage. Redoxomics is the most fascinating new measure to address lung damage due to oxidative stress. The new challenge is to use omics data to discover a set of biomarkers useful in diagnosis, prognosis, and formulating optimal and individualized neonatal care. The aim of this review was to examine the most recent evidence on the relationship between oxidative stress and lung diseases in preterm newborns. What is currently known regarding oxidative stress-related lung injury pathogenesis and the available preventive and therapeutic strategies are also discussed.

## 1. Introduction

**Figure 1:**
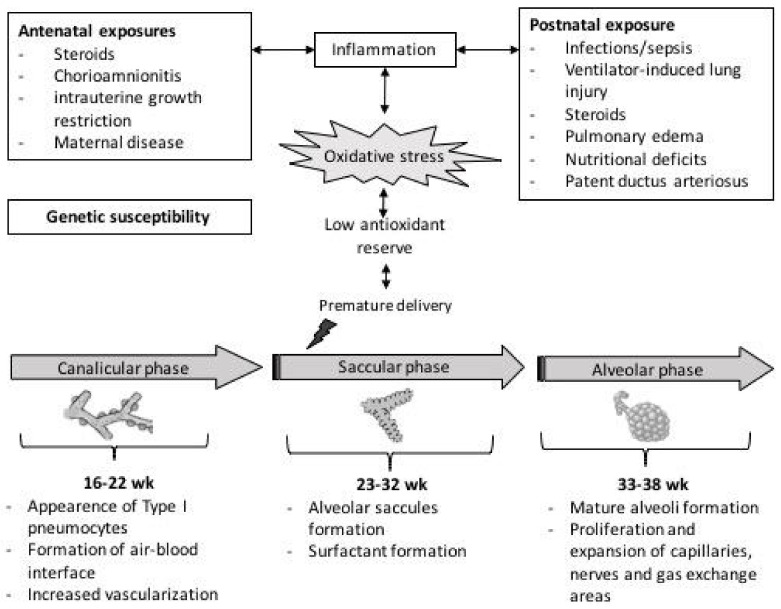
Schematic representation of mechanisms involved in free-radical mediated lung diseases following preterm delivery.

Pulmonary development begins early in intrauterine life and continues in the first years of life according to a sequence of events that can be summarized in five phases: embryonic, pseudoglandular, canalicular, saccular, and alveolar stages [1]. During the embryonic stage (4–6 weeks), lung development begins as an expulsion of the primary buds, from which the proximal portion generates the larynx and trachea while the distal end gives rise to the bronchi. The pseudoglandular stage (5–17 weeks) is mainly responsible for the generation of the bronchial tree up to the terminal bronchioles, with the formation of an arterial system, cartilage, and smooth muscle. During the canalicular (16–25 weeks) and saccular (24–32 weeks) stages, the process of differentiation between the conductive pathways and alveoli begins, and the gas exchange surface of the lungs expands significantly. Furthermore, at 24 weeks, the production of pulmonary surfactant starts up. Finally, in the alveolar phase (from 33 GA weeks to infancy), the last division of the saccules into alveoli occurs. The alveolar division process continues throughout childhood [1], Figure 1.

Preterm infants are born at a time when the lung is still in the embryological stage, especially if born before 30 weeks of gestation. Some features of the preterm lung, such as surfactant deficiency, structural abnormalities, and inadequate antioxidant defenses, can increase susceptibility to injury.

The factors that play a key role in the impairment of the pulmonary maturation process can be classified into intrauterine and postnatal causes [2]. Intrauterine factors include maternal disease, inadequate placental function, and exposure to antenatal steroids. Indeed, exposure to steroids on the one hand stimulates the production of surfactants and decreases inflammation, but on the other hand inhibits DNA synthesis and decreases lung repair and growth. Postnatal factors, however, are secondary to the baby’s poor growth, lung and systemic infections, and exposure to high concentrations of inspired oxygen that can generate oxygen free radicals.

There is growing evidence linking early exposure to oxidative stress (OS) with an altered lung development process making the lung more susceptible to a number of diseases typical of premature babies such as respiratory distress syndrome, bronchopulmonary dysplasia (BPD) and persistent pulmonary hypertension.

The aim of this review was to examine the most recent evidence on the relationship between oxidative stress and lung diseases in preterm newborns. What is currently known regarding oxidative stress-related lung diseases pathogenesis and the available preventive and therapeutic strategies are also discussed.

## 2. Oxidative Stress in Perinatal Period

It is now known that the delicate balance between reactive oxygen species (ROS) production and antioxidant defenses can be upset even before childbirth in major pregnancy-related disorders such as preeclampsia, chronic hypertension, obesity, infections, premature rupture of membranes (pPROM), and intrauterine growth restriction (IUGR) [3,4]. This could be explained by the fact that maternal factors cause an increase in inflammation, which in turn influences placental function and induces nutritional defects and hypoxic consequences on the fetus [5]. Moreover, a correlation has been highlighted between maternal oxidative stress (OS) and markers of oxidative stress measured in the umbilical cord blood of the newborn [6]. These markers have been associated with the development of several postnatal diseases, suggesting that intrauterine exposure to oxidative stress is a significant risk factor, especially in babies born preterm [7,8].

In the preeclamptic placenta, increased ROS production is responsible for the inflammatory state and endothelial dysfunction through the production of proinflammatory cytokines such as tumor necrosis factor (TNF)-α and other molecules with negative effects on endothelial cells. Furthermore, TNF-α causes an increase in endothelial permeability with consequent interstitial edema, which leads to further ischemia and production of ROS [9].

Maternal obesity is associated with metabolic alterations and redox balance dysregulation. Intensified inflammation and oxidative/nitrative stress are found in the placentas of obese women in association with placental dysfunction. Obese pregnant women have reduced antioxidant systems (superoxide dismutase (SOD), glutathione, ratio of oxidized glutathione to reduced levels of antioxidant vitamins) and increased levels of malondialdehyde (MDA), carbonyl proteins, and nitrites [10]. Maternal overweightness or obesity is associated with increased OS in the cord blood of offspring [11]. Studies have also shown reduced mitochondrial activity and reductions in adenosine triphosphate (ATP) in the placental tissue of obese women compared to normal pregnancies, suggesting that obesity alters the fetal–placental barrier [12].

Infections in pregnancy are associated with a higher incidence of BDP due to lung tissue exposure to proinflammatory cytokines and ROS. Inflammation causes impaired lung development. High levels of cytokines in the amniotic fluid (TNF-α, interleukin (IL)-8, IL-1β, IL-6) cause direct exposure of the fetal lung to proinflammatory cytokines that trigger a response from the innate pulmonary immune system, with the recruitment of neutrophils and macrophages and a simultaneous decrease in processes such as angiogenesis, morphogenesis, and cell development, leading to arrest of lung development [13].

Finally, studies evaluating the oxidative balance in small and large for gestational age (SGA and LGA, respectively) infants have shown an increase in circulating levels of carbonyl proteins and hydrogen peroxide (H_2_O_2_) in both groups [14].

Identifying high-risk pregnancies may allow for the prevention of further oxidative damage by early treatment of children with antioxidant drugs [8].

Premature infants are at risk of “oxygen radical disease” both because they are exposed to increased generation of ROS and because they have an ineffective antioxidant defense system [2,11]. Since antioxidant defense mechanisms progressively increase during gestation, premature birth interrupts both production processes and the passage of antioxidants from mother to fetus too soon [15].

The formation of ROS during the transition from fetal to neonatal life is due to complex physiological changes secondary to the different availability of oxygen between the intrauterine and extrauterine conditions [16,17]. Indeed, during intrauterine life, the fetus is in a hypoxic environment with a partial pressure of oxygen (PaO_2_) of approximately 20–25 mmHg [18]. At birth, with the onset of respiration, the oxygen concentration doubles. In this condition of increased oxygen availability, ROS production begins. Moreover, postnatal inflammation plays a key role in OS, because inflammatory cells release large amounts of ROS and proteases, resulting in cell damage, endothelial dysfunction, and overproduction of proinflammatory cytokines and free radicals [18].

Most ROS, such as the superoxide radical (O_2_^−^), H_2_O_2_, and the hydroxyl radical (OH^−^) are generated inside the mitochondria and tend to damage three cell substances, proteins, nucleic acids, and lipids, causing protein oxidation, DNA oxidation, and lipid peroxidation, respectively, with consequent damage to the membranes [19].

The oxidation of a protein occurs by oxidation of the side chains of amino acids, with alteration of the structure and therefore the function of the protein. The oxidation of DNA causes mutations or macroscopically damages the DNA itself and alters the chemical structure of the nitrogenous bases, forming new bases such as 8-oxyguanine or 5-hydroxymethyluracil [20].

Finally, lipid peroxidation of the plasma membrane and the membranes of intracellular organelles is caused by the reaction between free radicals and membrane lipids generating lipid peroxides, which, being reactive, propagate, causing extensive damage to the membranes.

The cell has several methods of metabolizing ROS, through enzymes, nonenzymatic proteins, oxidizable molecules, and trace elements. The enzymatic mechanisms are predominant. Enzymes such as SOD, catalase (CAT), and glutathione peroxidase are responsible for converting reactive oxygen species into less reactive and toxic products. Of all, SOD group enzymes, including manganese-dependent (MnSOD) and extracellular superoxide dismutase (EC-SOD), are the most powerful antioxidant enzymes [21]. MnSOD is a mitochondrial antioxidant enzyme that catalyzes the conversion of superoxide radicals to hydrogen peroxide, while EC-SOD is a Cu/Zn SOD secreted into the extracellular space that catalyzes the dismutation of two superoxide radicals into hydrogen peroxide and molecular oxygen. CAT is an antioxidant enzyme located in peroxisomes that catalyzes the breakdown of H_2_O_2_ into H_2_O and O_2_. Finally, the antioxidant system of glutathione consists of three functionally related enzymes: glutathione peroxidase, glutathione reductase, and glutathione S-transferase. The former reduces glutathione to convert H_2_O_2_ into oxidized glutathione; the latter, by restoring glutathione, reduces oxidized glutathione and peroxides and conjugates toxic electrophilic compounds to glutathione [20,21].

Nonenzymatic antioxidants such as glutathione, tocopherol, and ascorbic acid were evaluated by M. Matyas et al., who showed that their reduction was directly related to gestational age and oxidative stress [15].

Another molecule with antioxidant properties is melatonin [22]. This pineal hormone achieves its action both through a direct detoxification of ROS and indirectly by stimulating antioxidant enzymes. Furthermore, melatonin chelates the transition metals, which are involved in the Fenton and Haber–Weiss reactions, thus reducing the formation of the devastating toxic hydroxyl radical. [22,23]

## 3. Oxidative Stress and Respiratory Distress Syndrome

Respiratory distress syndrome (RDS), formerly known as hyaline membrane disease, is a disease of premature infants characterized by structural immaturity of peripheral airways and surfactant deficiency [18]. Male infants are more significantly affected by RDS than girls [24]. Male predisposition appears to be due to the inhibitory effect of androgens on lung maturation and surfactant production [25]. The surfactant is a complex mixture of phospholipids, neutral lipids, and specific proteins produced and secreted by type II alveolar cells to counteract alveolar collapse, with a potential antioxidant action [26].

The role of OS in RDS was demonstrated by the increase in ROS such as MDA, protein carbonyls, and 7,8-hydroxy2-deoxy guanosine (7,8-OHdG) [27]. Dizdar et al. demonstrated that in children with RDS, the increase in oxidant status against antioxidant mechanisms is associated in a proportional manner with greater severity and mortality [28]. Extremely premature and very low birth weight infants are more susceptible to rapid formation of free radicals, and therefore to RDS [24]. ROS lead to an increase in the permeability of the endothelium, resulting in the passage of polymorphonuclear leukocytes (PMNs) into the alveolar lumen and the release of cytokines, free radicals, and toxic nitrogen derivatives (RNS) that amplify the inflammatory process [18,29]. Indeed, high levels of ROS/RNS, myeloperoxidase, and oxidized-1-antitrypsin have been found in the bronchoalveolar lavage (BAL) fluid of patients with RDS [30]. Moreover, in infants with RDS, there are higher concentrations of MDA and H_2_O_2_ associated with a significant decrease in the activity of antioxidant enzymes [24]. From this perspective, oxidant/antioxidant status could provide a prognostic marker in newborn with RDS, helping to recognize high-risk infants [31,32]. Although it is now known that the balance between oxidizing and antioxidant factors plays a crucial role in the pathogenesis of RDS, there is a long way to go to achieve optimal therapeutic management, especially in newborn infants at high risk for impaired pulmonary function.

To date, the administration of endotracheal surfactants is the main etiopathogenetic treatment for RDS. Treatment with exogenous surfactant reduces surface tension, determines an increase in antioxidant substances, and counteracts the accumulation of intra-alveolar ROS [30]. Indeed, because exogenous surfactants carry high concentrations of both SOD and CAT activity, a reduction in oxidized glutathione levels has been demonstrated in BAL of infants treated with surfactant [33].

Moreover, another study showed that aerosol administration of SOD improved alveolar development in baboons with RDS, reducing the onset of bronchopulmonary dysplasia [34].

Because of its adsorption and diffusion properties, surfactant is also a potential vehicle for drugs targeting the airways, including synthetic glucocorticoids such as budesonide [35]. In animal models, the addition of budesonide to the surfactant has shown beneficial anti-inflammatory effects [36].

In two subsequent randomized controlled trials (RCT), Yeh et al. tested a budesonide–surfactant blend for the prevention of BPD in preterm infants with RDS, showing a noticeable reduction in BPD [37,38]. A large RCT on surfactant supplemented with budesonide for the prevention of BPD is currently underway (SASSIE study: ClinicalTrials.gov NCT02907593, accessed in September 2016).

## 4. Oxidative Stress and Chronic Lung Disease

Bronchopulmonary dysplasia is a chronic lung disease (CLD) diagnosed when premature infants still require oxygen therapy at 28 days of life or at 36 weeks of gestation. BPD is a consequence of the association of two events: early interruption of the lung development process and postnatal lung injuries. The pulmonary development process can be interrupted in the canalicular or saccular phase if delivery occurs at 24–26 or 26–32 weeks of gestation, respectively. In the canalicular phase, the differentiation of the epithelia takes place, which leads to the formation of the airways, while in the saccular phase, the morphogenesis of the branches ends and alveolarization begins [39]. Consequently, preterm birth causes a structural alteration of the lung, which is more severe the lower the gestational age.

After birth, OS-induced pulmonary and vascular remodeling underlies BPD [40]. ROS, in fact, alter the molecular pathways involved in lung development, leading to impaired alveolarization, pulmonary microvascular remodeling, smooth muscle hyperplasia, and moderate fibrosis [32,41]. First, OS causes excessive apoptosis of type II pneumocytes, which play a key role in regulating lung tissue growth, maturation, and repair processes and lung fluid homeostasis [42]. Furthermore, OS induces an imbalance of mesenchymal–epithelial signaling that leads to the transdifferentiation of pulmonary alveolar lipofibroblasts into myofibroblasts [43].

Lipofibroblasts are homologues of adipocytes, which protect the alveolus from oxidative damage by regulating, through the secretion of leptin, the synthesis of surfactant by type II pneumocytes [44,45]. Lipofibroblast differentiation is induced by the peroxisome proliferator-activated receptor gamma (PPARγ) [46]. In presence of factors that cause deranged mesenchymal–epithelial signaling, pulmonary lipofibroblasts lose their lipogenic phenotype and transdifferentiate into a myogenic phenotype (MYF) [43]. MYF are unable to maintain lung epithelial cell growth and differentiation, resulting in the typical alveolarization failure observed in BPD. Studies on cellular and animal models showed that PPARγ agonists can not only block but reverse the lipofibroblast on MYF transdifferentiation, preventing and potentially reversing progression in CLD. Indeed, administration of PPARγ agonists, such as curcumin, to neonatal rat models has been shown to inhibit myofibroblast differentiation and improve neonatal lung maturation [47].

Regarding vascular remodeling, studies have shown that vascular endothelial growth factor receptor 2 (VEGFR2) is essential for preserving normal vascular and pulmonary structure, but it is deficient in preterm infants who develop BPD [48,49]. Moreover, while the use of VEGF antagonists in neonatal rats resulted in a significant alteration of alveolar development, upregulated VEGF expression prevented lung injury in preterm rabbits [50].

The use of postnatal glucocorticosteroids for the prevention and alleviation of BPD is a controversial topic [51]. Indeed, the administration of dexamethasone during the first week of life was associated with both a shorter time of mechanical ventilation and an improvement in the outcome of BPD, as well as an increased risk of cerebral palsy. However, administering low-dose dexamethasone to newborn over one week of age on mechanical ventilation was associated with a higher likelihood of extubation without adverse effects on brain outcome [52].

Furthermore, some authors have shown that even low-dose hydrocortisone treatment in the first week of life is associated with a small but significant improvement in the diagnosis of BPD, especially in children exposed to chorioamnionitis, but long-term pulmonary benefits have yet to be determined [53,54].

Oxidative insult is a salient part of lung injury that acts initially as acute inflammatory damage in RDS and then as a chronic and structural scarring leading to the development of BPD [55]. In a recent study, 8-hydroxy-2-deoxyguanosine (8-OHdG) concentrations were measured in serum and tracheal aspiration samples from very low birth weight preterm infants. The authors found significantly higher 8-OHdG concentrations in children who subsequently developed BPD at both day 1 and day 28 of life [56]. Understanding the mechanisms of action of oxidative stress plays an important role in improving the prognosis of BPD by guiding the clinical use of oxygen and antioxidant therapies. Among the antioxidants, vitamins A and E were evaluated as to whether their use could inhibit lipid peroxidation induced by ROS and eliminate ROS. However, a Cochrane meta-analysis suggested that vitamin A supplementation did not significantly improve neurological and lung development at 18–22 months of corrected gestational age [8]. Similarly, vitamin E supplementation did not demonstrate a reduction in the incidence of BPD either [8].

In recent years, biomarkers that can accurately predict lung damage in newborns have been studied in order to distinguish children at risk of chronic lung disease early on. In this regard, the determination of 8-OHdG in urine appears to be able to identify healthy preterm infants from those with BPD, and among the latter, those with mild BPD from those with moderate to severe BPD [57]. However, because of the complexity and multifactoriality of oxidative stress disease, it appears that it is not enough to search for a single biomarker; rather, biomarker panels should be used [58].

Redoxomics is the most fascinating new measure to address lung damage due to free radical insult. Omics technology provides clinically useful information from genomics, epigenomics, proteomics, and metabolomics. The challenge is to use omics data to discover a set of biomarkers useful in diagnosis, prognosis, and formulating optimal and individualized neonatal care. The role of this integrative approach in BPD has been emphasized by recent studies confirming that OS can start very early in life and/or even prenatally [59,60,61]. Piersigilli et al. demonstrated an increase in acylcarnitines C16-OH and C18:1-OH in neonates who developed BPD. Acylcarnitines are released during β-oxidation of fatty acids, suggesting an increased presence of oxidative stress in BPD patients as one of the pathophysiological mechanisms of the disease. It is therefore crucial to protect the most predisposed preterm infants from oxidative injury.

## 5. Oxidative Stress and Persistent Pulmonary Hypertension of the Newborn

Persistent pulmonary hypertension of the newborn (PPHN) is caused by an abnormal transition at birth that results in high pulmonary vascular resistance (PVR), right-left extrapulmonary shunt of the deoxygenated blood, and severe hypoxemia [62]. Several studies showed that antenatal exposure to oxidative stress, fetal growth restriction, and postnatal stresses, such as hyperoxia, aggressive ventilation, and sepsis, may amplify the damage on pulmonary vascularization [63,64]. It is now well known that pulmonary vascular tone depends on the balance between ROS and antioxidant enzymes. In fact, physiologically, the nitric oxide (NO) produced by endothelial NO synthase (eNOS) stimulates vasorelaxation by activating soluble guanylate cyclase (sGC), which in turn produces cyclic guanosine monophosphate (cGMP), while phosphodiesterase type 5 (PDE5) degrades cGMP, compromising vasorelaxation [65]. Stressful events worsen PPHN by reducing the expression of vascular tone modulators and the response to pulmonary vasodilators, resulting in a decrease in pulmonary vessel density and thickening of the pulmonary arteriolar walls [66,67].

During the transition to extrauterine life, the physiological increase in PaO2 promotes the endogenous release of NO and therefore vasodilation. However, because PPHN is generally associated with severe hypoxemia, neonates are often exposed to high oxygen concentrations [68].

Studies have shown that chronic exposure to hyperoxia induces expression of NADPH oxidase (Nox) in the lungs of newborn mice and increases the cytosolic activity of ROS and PDE5 in smooth muscle cells of lambs’ pulmonary arteries [69,70]. ROS not only potentiate pulmonary vasoconstriction by reducing the ability of pulmonary artery endothelial cells to respond to NO, interfering with NO/GMP pathway enzymes, and decreasing cGMP levels, but lead to surfactant inactivation [71,72].

Furthermore, hyperoxia has been shown to cause mitochondrial dysregulation and genetic reprogramming in the right ventricles of premature rats and stimulate PASMC proliferation in newborn sheep [73,74]. These two mechanisms increase vascular hypertrophy and dysfunction, which are essential for the initiation and maintenance of PPHN.

Current therapeutic strategies are based on the restoration of all these mechanisms that are altered by oxidative stress, such as the administration of inhaled nitric oxide, sildenafil, bosentan, milrinone, and prostaglandins, which act as vasodilators, in addition to glucocorticoids, which play a role in reducing inflammation. Among commonly used drugs, sildenafil has been shown to reestablish vascular cGMP signaling and reduce right ventricular hypertrophy in a mouse model of hyperoxia-induced PPHN [74].

Given the fundamental role of oxidative stress in the pathogenesis of PPHN, antioxidant drugs may be effective in limiting ROS production in oxygen-ventilated PPHN infants.

In this regard, it is now known that SOD is insufficient in the pulmonary vascular system of premature babies. Since deficiency in this enzyme facilitates endothelial dysfunction and reduces vasodilation induced by nitric oxide, its administration has been evaluated for more than a decade in order to improve the outcome of lung disease [75]. Animal studies showed that intratracheal administration of antioxidants and recombinant human SOD (rhSOD) decreased ROS, increased eNOS expression, and improved NO-mediated vasodilation in neonatal lamb models of PPHN exposed to 100% O_2_ for 24 h [76,77]. Moreover, a recent study in lamb models showed that addition therapy with the administration of superoxide dismutase to iNO reduced oxidative stress and restored eNOS coupling [77]. Although the effect of rhSOD in reducing oxidative stress and restoring coupling of eNOS is now recognized, the evidence is limited to animal models of PPHN, as its use has never been studied in humans. However, these recent findings set the stage for further research perspectives on an interesting alternative in the treatment of the neonatal population.

In addition, some antioxidants such as tetrahydrobiopterin, N-acetylcysteine, apocynin, and ascorbic acid have also been shown in animal studies to reduce intracellular ROS and achieve better NO bioavailability, but their benefit has not yet been confirmed in human infants [78].

To date, clinical trials on the efficacy of antioxidant therapy in the treatment of ROS-induced neonatal pulmonary hypertension have had limited results. Therefore, subsequent studies will need to be more precisely directed at cellular and subcellular targets that control oxidative balance.

## 6. Oxidative Stress and Ventilation-Induced Lung Injury

The most common reason for respiratory support is RDS. However, mechanical ventilation can impair lungs, inducing CLD, because preterm lung volumes are small, the lung matrix is not fully developed, and surfactant can be lacking.

Historically, resuscitation in the delivery room was performed with 100% oxygen. However, several studies have shown that high concentrations of oxygen (FiO_2_) are not only no more effective than low FiO_2_ but are associated with increased oxidative stress [79,80,81,82]. Hyperoxia damages the barrier of the airway epithelium and increases lung permeability, fluid congestion, and the release of inflammatory mediators that attract inflammatory cells into the immature lung [83]. In turn, activated macrophages and neutrophils produce ROS and proinflammatory cytokines and chemokines, supporting lung injury [84]. Indeed, elevated levels of proinflammatory mediators were found in tracheal aspirates and bronchoalveolar lavage fluid from premature infants [85]. The latest European guidelines suggested using initial FiO_2_ of 21–30%, gradually increasing it if necessary to reach an adequate SpO_2_ [86]. In addition to oxygen toxicity, volutrauma is the most important reason for lung damage, the release of proinflammatory cytokines, and the production of free radicals. During resuscitation, the tidal volume (VT) is not monitored, risking a VT higher than necessary [32]. Pulmonary overdistension can worsen lung damage by inducing interstitial and alveolar edema, increasing epithelial and microvascular permeability, and attracting neutrophils and macrophages [87]. Previous studies showed a plasma increase in proinflammatory cytokines, such as IL-8, IL-1β and TNF-α, and a decrease in the anti-inflammatory cytokine IL-10 after only 2 h of ventilation [84,88,89,90]. Special modes of ventilation, such as high-frequency oscillatory ventilation (HFOV) and the volume guaranteed (VG) method, may reduce the pressure and volume of gas delivered to the lungs. HFOV reduces volutrauma by using a small VT, maintaining nearly constant alveolar pressure, and optimizing lung volume by adjusting mean airway pressure. GV integrates various pressure-controlled ventilation modes and provides a fixed tidal volume, decreasing positive inspiratory pressure (PIP) based on improvements in compliance, endurance, and spontaneous activity.

Finally, when possible, noninvasive ventilation (NIV), such as nasal intermittent positive pressure ventilation (nIPPV) with the use of nasal continuous positive airway pressure (nCPAP), should be preferred. The nCPAP can be used from the delivery room to support first spontaneous breaths, reducing the risk of supplemental oxygen and intubation and improving long-term respiratory outcome [91,92]. A simple and effective lung protection strategy is to keep the lung “open” or recruited by finding the optimal level of PEEP for each patient, typically between 5 and 9 cm H_2_O, to maintain lung expansion and prevent end-expiratory alveolar collapse [86].

The choice of lung protection strategies should be elaborated based on the pathophysiology of the disease and pulmonary maturity, achieving a balance between the gas exchange targets and the potential toxicities of the treatments.

## 7. Conclusions

The complex mechanisms by which OS contributes to neonatal lung injury are not yet fully understood.

Free radicals and ROS are toxic oxygen derivatives that destroy lipids, proteins, and DNA inside cells. Since preterm infants are highly sensitive to oxidative stress, strategies to limit ROS production need to be activated. Although oxidative stress is difficult to characterize, biomarker assay can be useful in the early identification of children at high risk of tissue damage, allowing targeting and predicting the potential efficacy of therapies. To date, the most important approach to limiting OS-related lung damage has been to use gentle ventilation with the lowest possible FiO_2_ to maintain adequate SpO_2_, a small VT, and a level of PEEP high enough to keep the lung “open” and avoid alveolar collapse.

In addition to the ventilation strategy, antioxidant drugs are critical for regulating inflammation and protecting against ROS-induced injury.

Studies are currently underway to evaluate potential therapeutic antioxidants in human preterm infants. Redoxomics is the most fascinating new measure to address lung damage due to free radical insult, paving the way to new antioxidant strategies. It is desirable that ongoing studies yield positive results to definitively solve a major clinical problem in extremely low birth weight infants.

## Data Availability

Not applicable.
